# A High Temperature Capacitive Humidity Sensor Based on Mesoporous Silica

**DOI:** 10.3390/s110303135

**Published:** 2011-03-14

**Authors:** Thorsten Wagner, Sören Krotzky, Alexander Weiß, Tilman Sauerwald, Claus-Dieter Kohl, Jan Roggenbuck, Michael Tiemann

**Affiliations:** 1 Department of Chemistry, Faculty of Science, University of Paderborn, Warburger Str. 100, D-33098 Paderborn, Germany; E-Mail: michael.tiemann@upb.de; 2 Max-Planck-Institute for Solid State Research, Nanoscale Science Department, Heisenbergstr. 1, D-70569 Stuttgart, Germany; E-Mail: s.krotzky@fkf.mpg.de; 3 Institute for Applied Physics, Justus Liebig University, Heinrich-Buff-Ring 16, D-35390 Giessen, Germany; E-Mails: alexander.weiss@ap.physik.uni-giessen.de (A.W.); tilman.sauerwald@ap.physik.uni-giessen.de (T.S.); kohl@physik.uni-giessen.de (C.-D.K.); 4 Institute of Inorganic and Analytical Chemistry, Justus Liebig University, Heinrich-Buff-Ring 16, D-35390 Giessen, Germany

**Keywords:** capacitive sensor, humidity, high temperature, mesoporous silica

## Abstract

Capacitive sensors are the most commonly used devices for the detection of humidity because they are inexpensive and the detection mechanism is very specific for humidity. However, especially for industrial processes, there is a lack of dielectrics that are stable at high temperature (>200 °C) and under harsh conditions. We present a capacitive sensor based on mesoporous silica as the dielectric in a simple sensor design based on pressed silica pellets. Investigation of the structural stability of the porous silica under simulated operating conditions as well as the influence of the pellet production will be shown. Impedance measurements demonstrate the utility of the sensor at both low (90 °C) and high (up to 210 °C) operating temperatures.

## 1. Introduction

Reliable detection of relative humidity in gases is an important issue in numerous fields, including industrial processes, meteorology, and everyday domestic life. Humidity sensors are based on a wide range of working principles and technologies [1–4]. Among these, capacitive sensors are the most common because they are both inexpensive and their detection mechanism is very specific to humidity [5,6]. Typically a hygroscopic polymer or ceramic is placed between the adjacent plates of a capacitor; adsorption of water from the gas phase results in changes of the capacity [3,7].

However, for many industrial processes a high durability and resistance to harsh environments are of particular importance. For example, industrial branches with high energy saving potential, such as wood drying or textile processing, demand sensors with high tolerance to corrosive/oxidizing species or contaminants with high molecular weight at high temperature (>200 °C). These requirements often exclude the utility of conventional sensor materials [3,4] as they are usually unstable under these conditions. Furthermore humidity sensing becomes increasingly challenging at high temperatures since sensors respond to changes in relative (rather than absolute) humidity. The relative humidity at high temperatures is generally very low, since the saturation vapor pressure of water increases exponentially with the temperature. Hence, for high-temperature humidity sensing extremely sensitive devices are necessary. Mesoporous silica devices shows superior properties in this respect due to their temperature stability, chemical inertness, and the option to modify their hygroscopy by pore-wall decoration. Here we report a sensor device based on ordered, mesoporous silica. We show humidity-sensing data up to 210 °C.

## 2. Experimental Section

### 2.1. Synthesis and Characterization of Mesoporous Silica

Mesoporous SBA-15 silica [8] was synthesized using Pluronic P-123 block-copolymer as a structure director. P-123 (Sigma, 12.0 g) was mixed with water (360 g) and HCl solution (32%, 43.0 g). After addition of tetraethyl orthosilicate (TEOS; Merck, 24.0 g) the mixture was stirred at 35 °C for 24 h. The resulting gel was transferred to a Teflon-lined autoclave and kept at 60 °C for 24 h. The solid product was filtered off, washed with water, and calcined at 550 °C for 6 h (heating rate 2 °C min^−1^).

Powder X-ray diffraction (XRD) was carried out on a “Panalytical X'Pert Pro” diffractometer (filtered Cu-Kα radiation, 40 kV, 40 mA). Nitrogen physisorption was conducted at −196 °C on a Quantachrome Autosorb 6; samples were degassed at 120 °C for 24 h prior to measurement. Specific surface areas were calculated from the adsorption data in the relative pressure interval from 0.05 to 0.2 using the BET method [9]. Total pore volumes were estimated from the quantity of nitrogen adsorbed at a relative pressure of 0.98. Pore size distributions were calculated from the desorption branch by the BJH method [10].

### 2.2. Sensor Device Preparation

**Figure 1.**
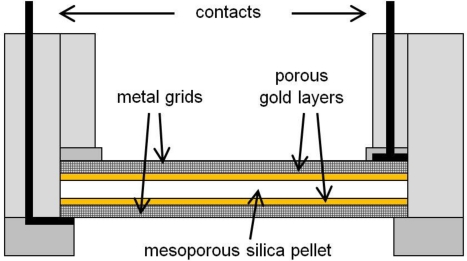
Schematic drawing (cross section) of the sensor housing used for the capacitance measurements. The mesoporous silica pellet is coated with macroporous gold layers on both faces which serve as the capacitor plates; they are contacted via metal grids.

A plate-type capacitor geometry was chosen for the capacitance measurements. Pellets of 23 mm diameter were made from mesoporous silica powders by using a MB 100400L hydraulic molding press (Schmidt Maschinentechnik, maximum pressure 1,000 kN). The thickness of the pellets was in the range of 0.5 to 1.5 mm. A pressure of *ca*. 20 kN/cm^2^ was found to be optimal for the stability of the pellet (see Results section). Pellets were coated with gold by vapor deposition using a Tectra Mini-Coater with quartz crystal microbalance. The gold forms porous layers to serve as electrodes, keeping silica accessible to water vapor; an optimum thickness for each gold layer was found to be 50 nm (see Results section). The gold layers were investigated by scanning electron microscopy (SEM) using a HREM EDX Leo Gemini 982 microscope. The shape of the electrodes was controlled by shadow masking. The gold-coated pellets were mounted into a specially designed sensor housing made of a high temperature stable polyimide (Vespel) as shown in Figure 1. For mechanical stabilization and to contact the gold electrodes the pellet was clamped in between two metal grids that are contacted to coaxial cables connecting the measuring electronics.

### 2.3. Humidity Measurements

**Figure 2.**
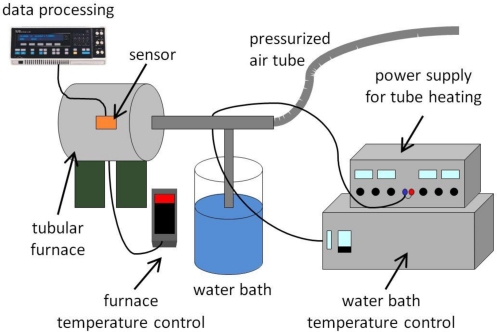
Scheme of the humidity measurement setup at temperatures above 95 °C. The humidity is controlled by the water bath temperature (see text).

Humidity measurements were carried out with two different setups. For low-temperature measurements up to 95 °C an environmental chamber (SB22/300/40, Weiss Klimatechnik) was used. Up to this temperature the absolute humidity in the chamber can be regulated from 0 to 98%. For high-temperature measurements a specially designed setup was used (Figure 2). Pressurized air is saturated with water using a temperature-regulated water bath. The saturated stream is then transferred into a tube oven where the sensor is heated to temperatures up to 250 °C through temperature-regulated transfer lines to prevent condensation. The humidity is set by controlling the temperature of the water bath.

In both cases, low and high temperature, an impedance analyzer (Solartron Analytical Si 1260) was used to record the changes in capacitance of the sensor at different frequencies. For low-temperature measurements a commercially available digital humidity sensor was used as a reference (Sensirion SHT11 [11]). This sensor is only specified up to a temperature of 125 °C; above this temperature damage to the polymer used as dielectric layer cannot be excluded.

## 3. Results and Discussion

### 3.1. Structural Properties of Mesoporous Silica

**Figure 3.**
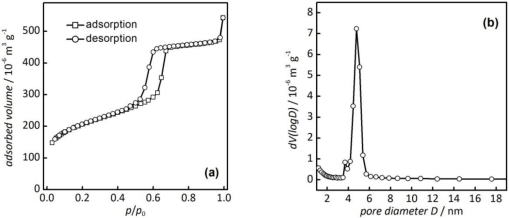
**(a)** Nitrogen physisorption isotherm and **(b)** pore diameter distribution of mesoporous SBA-15 silica.

The nitrogen physisorption isotherm of the mesoporous SBA-15 silica material [Figure 3(a)] shows a steep increase of adsorbed nitrogen at a relative pressure of *ca. p*/*p*_0_ = 0.6–0.7 in the adsorption branch and a hysteresis in the desorption branch.

This is characteristic for materials with uniform, cylindrical mesopores; the isotherm/hysteresis shape is classified by IUPAC as type-IV/H1 [12]. The average pore diameter is 4.8 nm, as shown in the pore diameter distribution plot (BJH method [4]) in Figure 3(b). This value is typical of SBA-15 silica synthesized at 60 °C. From the adsorption isotherm a specific surface area (BET method [3]) of 741 m^2^/g and a total pore volume of 0.710 cm^3^/g are obtained; all values are listed in Table 1.

**Figure 4.**
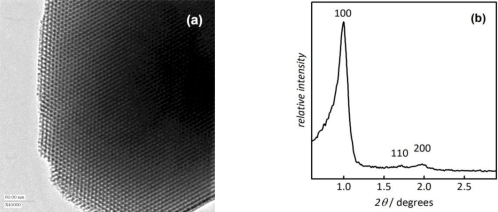
**(a)** Transmission electron microscopic (TEM) image and **(b)** low-angle powder X-ray diffraction (XRD) diagram of mesoporous SBA-15 silica.

The periodic order of the mesopores is apparent from the transmission electron microscopic (TEM) image [Figure 4(a)] which shows a view along the axis of the linear, cylindrical pores. The pores are arranged in a two-dimensional hexagonal symmetry. This is also reflected in the low-angle region of the powder X-ray diffraction (XRD) diagram [Figure 4(b)] which shows three characteristic peaks indexable to the *p*6*mm* space group.

### 3.2. Sensor Device

As described above, the general principle of a capacitive humidity sensor is based on the humidity-dependent change of the dielectric constant of the porous medium which is located between the adjacent electrodes of the capacitor. This change originates from the physical adsorption of increasing amounts of water in the porous medium with increasing (relative) humidity. As a result, a change in the capacitance (or impedance, respectively) becomes a measureable parameter for humidity.

In order for the mesoporous silica to serve as the dielectric medium a sensor device had to be fabricated in which a defined amount of silica is placed between adjacent plate-like electrodes. The electrodes must have a defined size and distance to each other. At the same time, the silica needs to be easily accessible to water vapor. These requirements are fulfilled by forming the silica powder into a solid pellet and by applying porous gold layers at both faces of the pellet. These gold layers serve as the capacitor plates. They intrinsically exhibit porosity due to the gas-phase deposition method; their pores are wider than those in the silica pellet (see below) in order to not obstruct the accessibility of the pellet for water vapor, as will be demonstrated in the following.

To check the structural integrity of the porous silica material after being pressed into pellets, some of these pellets were ground in a mortar and the resulting powder was characterized again by N_2_ physisorption and XRD (data not shown). The results show that the pressure applied in the molding press (20 kN/cm^2^) does not significantly compromise the mesoporosity and periodic structural order of the sample. The specific surface area and total pore volume are slightly lower than before the molding press (see Table 1), but the sorption isotherms still show type-IV shape and a sharp pore size distribution around 4.8 nm.

Mesoporous silica of the SBA-15 type [1] is known to exhibit a high degree of temperature tolerance. This was verified by storing some of the samples at 220 °C under ambient air for three weeks and by investigating them afterwards by N_2_ physisorption and XRD. As expected, no significant change in porosity was observed; the specific surface area and total pore volume are listed in Table 1. However, long-term application of the sensor device at this temperature under real conditions will likely include harsher environments, such as corrosive species in the gas phase. Therefore, the structural integrity of the porous silica material will have to be subject to further investigation.

For the preparation of the macroporous gold layers two requirements had to be observed: (i) The layers must be continuous and have a well-defined area for the calculation of the base capacitance. (ii) The layers must exhibit high porosity to allow diffusion of water vapor into (and out of) the dielectric layer. Both requirements can be controlled by varying the thickness of the gold layers. On the one hand only a sufficiently thick layer will be continuous and therefore exhibit defined conductivity as required. On the other hand, if the layers are too thick, then the porosity will naturally be too low. Hence, a compromise had to be found with respect to the gold layer thickness. This was accomplished by conductivity and SEM measurements. For the conductivity measurement the gold layer was contacted at opposite sides with a maximum electrode distance of 21 mm. To minimize the influence of contingent inhomogeneities at the contacts each measurement was repeated after changing the contact points.

**Figure 5.**
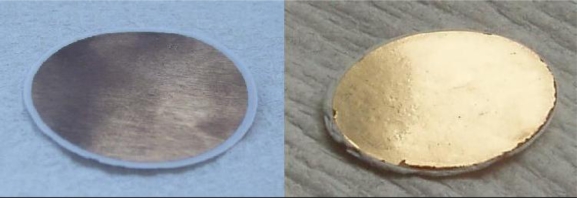
Two pellets (23 mm in diameter) of mesoporous silica coated with gold layers of 10 nm (left) 50 nm (right) thickness.

**Figure 6.**
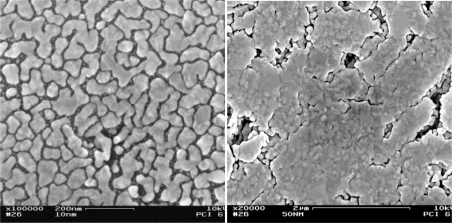
Scanning electron microscopic (SEM) images of the gold layers on mesoporous silica pellets; the layers have a thickness of 10 nm (left) and 50 nm (right), respectively.

In the following, two gold layers with thicknesses of 10 nm and 50 nm, respectively, will be compared exemplarily. Figure 5 shows photographs of these two samples. The thicker layer appears like macroscopic gold with a yellow color and metallic gloss; the thinner layer has a copper-like color with low degree of gloss. The conductivity measurements showed that only the thicker layer exhibits a sufficiently high electronic conductance; the intra-layer resistance measured at a maximum electrode-to-electrode distance of 21 mm (which is the diameter of the pellet) was below 10 Ω. For the thinner gold layer a high resistance above 20 MΩ was measured which means that this layer cannot serve as a capacitor plate. These findings can be explained by electron microscopic (SEM) investigation of the layers; Figure 6 shows micrographs of both specimens. The thin layer consists of small islands of gold (*ca.* 100 nm in size) which are poorly interconnected with each other. (Due to the low interconnectivity this layer could be measured by SEM only at regions close to the contact between sample and sample holder.) The thicker layer shows larger islands with good interconnectivity. At the same time large pores are visible which ensure efficient diffusion of water vapor through the gold layers. Hence, a gold layer thickness of 50 nm was chosen for the device.

### 3.3. Low-Temperature Humidity Measurements

**Figure 7.**
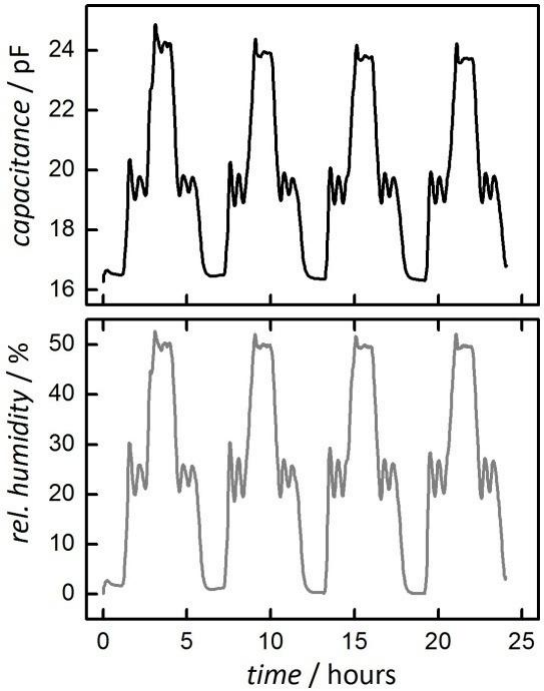
Change of capacitance of a sensor based on mesoporous silica (top) upon changes in the relative humidity between 0% and 50% at 90 °C and at a measurement frequency of 200 kHz. The ‘rel. humidity’ (bottom) is measured by a SHT11 reference sensor [4] which directly delivers the relative humidity by a digital bus. The humidity cycle was repeated four times (see text).

Figure 7 shows the result of a humidity measurement at 90 °C with a relative humidity profile in four steps (1 h 0%, 1 h 25%, 1 h 50%, 1 h 25%). This cycle was repeated four times to investigate the stability and reproducibility of the measurement.

The graph shows the change of capacitance of the sensor prepared with mesoporous silica (top) as well as the results from a SHT11 sensor (bottom) which serves as the reference [4]; its signal is plotted here as ‘relative humidity’. The measurement frequency for the silica-based sensor was 200 kHz. For commercially available sensors a frequency in the range of 35 kHz is usually applied; details regarding the influence of the measurements frequency will be discussed later.

Fluctuations in both signals, especially at 25% rel. humidity, are caused by the adjustment of the climatic chamber. The signal from the silica-based sensor shows a good reliability over the entire measurement period of 24 h. The capacitance shows a strong change of *ca*. 8 pF from 0% to 50% rel. humidity with a signal resolution comparable to that of the commercial reference sensor.

**Figure 8.**
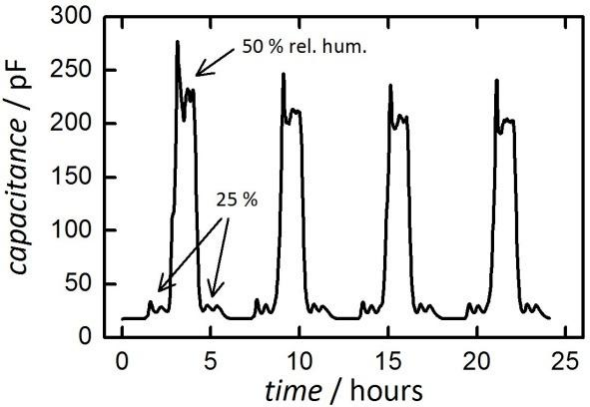
Results obtained from the same measurement conditions as in Figure 7 (0%, 25%, 50% rel. humidity; 90 °C), but with a low measurement frequency of 1 kHz. The non-linear increase of capacitance with rel. humidity is explained in the text.

To point out the above-mentioned influence of the measurement frequency on the sensing results Figure 8 shows data from a measurement at 90 °C under identical conditions, but with a low frequency of only 1 kHz. At a relative humidity of 25% the signal is comparable to that at high frequency. At 50% relative humidity, however, a much stronger, non-linear increase in capacitance is observed. This is caused by a misinterpretation of the input signals of the impedance analyzer by applying a wrong equivalent circuit. The strong increase is due to an ionic surface conductivity caused by proton transport (similar to the Grotthuss mechanism in liquid phase) [3]. This ionic conduction leads to the formation of a space charge layer at the ion-blocking gold electrodes which, in turn, leads to an increase in the overall capacitance but not of the capacitance of the dielectric.

### 3.4. High-Temperature Humidity Measurements

**Figure 9.**
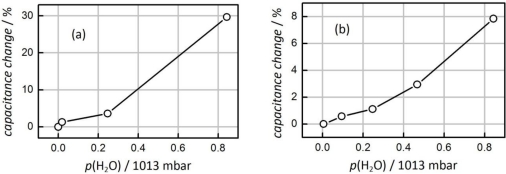
Change of capacitance of a sensor based on mesoporous silica **(a)** at 150 °C and **(b)** at 210 °C. Data were recorded using a measurement frequency of 200 kHz.

In the preceding section it was proven that the sensor based on mesoporous silica is suitable for the detection of 0% to 50% relative humidity at a relatively low temperature of 90 °C. For this temperature region other commercial capacitive humidity sensors are available (such as the SHT11 reference sensor), and the silica-based sensor delivers similarly reliable results. Measurements at higher temperatures (e.g., 210 °C) are substantially more challenging, since relative humidity is generally very low at these temperatures; in addition most organic sensor materials are not stable, therefore commercial capacitive humidity sensors are not applicable at high temperatures. The sensor presented here has a high thermal stability which makes it generally eligible for high-temperature applications. Figure 9 shows the change of capacitance measured at 150 °C and at 210 °C, respectively. In both cases humidity-related changes in capacitance are clearly observed. At 150 °C a change in relative humidity from 0% to 19% (corresponding to 854 mbar H_2_O) leads to a change in capacitance of e.g., 30%. At a temperature of 210 °C a change in relative humidity from 0% to 4.5% (corresponding to 854 mbar H_2_O) leads to a change of 8%. The sensor shows a near-linear response behavior to relative humidity with only little influence of the temperature. The sensor response (*i.e.*, change in capacitance divided by change in relative humidity) is 1.6 at 150 °C and 1.7 at 210 °C. The data confirm that the sensor is sufficiently sensitive, e.g., for humidity control in drying processes.

## 4. Conclusions

A capacitive sensor design based on a mesoporous silica pellet as the dielectric with macroporous gold layers as electrodes is capable of measuring humidity at low (<90 °C) and high temperatures (up to 210 °C). Measurement frequencies of 200 kHz were found to be suitable to minimize the influence of leak currents caused by ionic surface conductivity. A linear response curve in the high-temperature region (150 °C–210 °C) and a consistent response make the sensor eligible for high-temperature applications such as industrial drying processes. The regular porosity of the mesoporous silica was proven to tolerate long-term treatment comparable to operating conditions and processing during sensor preparation.

## Figures and Tables

**Table 1.** Porosity parameters of mesoporous silica.

**Silica Sample**	**Average Pore Size (nm)**	**Total Pore Volume (cm^3^/g)**	**Specific Surface Area (m^2^/g)**
as synthesized	4.8	0.710	741
after storing at 220 °C	4.8	0.700	723
pressed pellet	4.8	0.587	548
